# Reveiws

**Published:** 1800-12

**Authors:** 


### CRITICAL RETROSPECT

OF

### MEDICAL AND PHYSICAL LITERATURE.

### [FOREIGN AND DOMESTIC.]

*Samuelis Thomæ Soemmering Tabula Bafeos Encephali; Francofurti ad Mœnum,* 1799, *impenfis Auctoris, with two plates, in fmall folio, price* 2 *florins* 45 *kreutzer, or about* 5*s.*

Few anatomifts have acquired fuch great and unqueftionable praife for the anatomy and phyfiology of the human brain as the celebrated Mr. Soemmering; to him we are indebted for a more accurate knowledge and diftribution of the origin of the nerves, of which he has for that purpofe given a moft excellent table, reprefenting the *bafis encephali*; and what is a certain proof of its excellence, Mr. Vicq d'Azyr had it afterwards copied in his valuable work. He was the firft who difcovered the third and fourth fubftance, *(fubftantia fubflava et nigricans).* He firft proved the fand in the brain to belong to the natural ftate of it; he firft propofed the very interefting and inconteftible principle, that man has the greateft brain in proportion to the thicknefs of his nerves; and laftly, he conceived the moft ingenious, though hypothetical idea of of the vapour or water in the ventricles of the brain being the feat or organ of the foul. After having given fo many trials of his fuccefsful endeavours in enlightening the anatomy and phyfiology of the brain, and of his fkill in reprefenting it, a new Tabula Bafeos Encephali muft naturally raife the expectations of every anatomift, particularly, as that which he publifhed twenty years ago feemed to be matchlefs. Mr. Soemmering, however, intended to give here a moft finifhed reprefentation of the bafis encephali. "Tabulam, (he fays) quantam in me fuit, omnibus numeris abfolutam;" and at the fame time, to difcufs again fome of his former difcoveries, and fupport them with new arguments. For this purpofe, a brain which he had taken out of the fkull of a boy of three years old, feemed to be perfectly calculated, by having all parts complete and vifible; and this he accordingly got drawn under his infpection by Mr. Köck, a very excellent artift. Befides, as the foft brain is very apt to fall together, and by that means to lofe its natural form, the fkull from which it had been taken was filled with plafter of Paris, and in this way the true form, though rudely, was reprefented; after which it was corrected and accurately copied in the drawing. The artift has been particularly careful with refpect to the true fituation of the nerves. The table is engraved by *P. M. Alix* at Paris, in aquatinta, a manner particularly proper for the reprefentation of fuch foft parts as the brain, by marking the foftnefs and unperceivable tranfition of one part to another much better than any other method. Although it is rather difficult to preferve in this manner the accurate fketches of fingle parts, yet this is performed by the artift in a moft fuperior way. All the inequalities and windings of the furface of the cerebrum, and each nervous filament are fo mafterly and diftinctly figured, without hurting the foftnefs of the whole, that this plate may be confidered as a chef d'œuvre, as well in point of art as in accuracy and precifion. It bears not only an impartial comparifon with the excellent tables of Vicq d'Azyr, but in many refpects furpaffes them, and particularly the windings and impreffions are here better and more faithfully reprefented. The infertion and fituation of fome of the nerves, viz. the fourth, are by no means accurately enough figured in the tables of Vicq d'Azyr; and on a clofe examination of Soemmering's tables with thefe, they will be found much fuperior. There are about 300 good copies ftruck off; the weaker impreffions are marked with letters, referring to the explanation, and two plates are therefore given with every copy of the publication, a good one, and a weaker print with letters. In the letter-prefs, the author farther difcuffes fome of the difcoveries he had already mentioned a long time ago. The firft *dogma* eftablifhed by him, is, *that man has the greateft cerebrum in proportion to the nerves arifing from it*; or, in other words, *man has the fmalleft nerves with the greateft cerebrum*; and in this difproportion of the mafs of the brain to the nerves, the reafon of his mental faculties is founded. This principle has been conftantly proved by the diffection of the brains of of different animals; and anatomists and physiologists of the first rank, Alex. Monro, Vicq d'Azyr, Blumenbach, &c. are so convinced of its truth, that it is now considered as a physiological canon, and a law of animal economy.

The second *dogma* is, that *the human cerebrum differs also from the brain of animals by the proportion its single parts bear to one another.* From a comparison of this table with the baseos encephali of animals, it appears that the human cerebellum is proportionably much smaller than that of animals, that the medulla spinalis is likewise much thinner, and that the windings of the brain are differently formed. The eminences and tubercles are also different from those of animals.

The third *dogma* refers to the largeness and property of the brain of a boy three years old, as is represented here, viz. to its being almost as big as the cerebrum of an adult, which will appear more evident by comparing it with the figures of *Vicq d'Azyr* and *Santorini.* It however differs from the brain of an adult by its substance being softer, its tunica arachnoides thinner; by the darker substantia corticalis, and whiter substantia medullaris; also, by the nerves being rounder, softer, more slender and fibrous; the olfactory nerves being thicker and shorter, &c. the auditory nerve only having the same size and structure.

The fourth *dogma,* first laid down by Mr. Soemmering, and confirmed in this table, particularly in the olfactory and auditory nerve of the left side, is this: *the nerves are conical,* i. e. the more a nerve removes from its origin, the more it increases in thickness.

These are the most interesting contents of this classic publication. It would have been necessary to translate it entirely, if we had intended to communicate every interesting idea it contains; particularly what Mr. Soemmering says of the difference of the brains of different animals, &c. but we hope that every anatomist will read and study it.

---

*Samuelis Thom. Soemmering, Icones Embryonum Humanorum*; Francofurti ad Moenum, 1799, 10 pp. with two plates in large folio, on vellum paper, price 9 florins, or about 18s.

In this publication Mr. Soemmering has given another instance of his great anatomical abilities in throwing a new light upon one of the most interesting parts of Anthropology; and this he has done in such a superior manner, as might be expected from such an able and ingenious anatomist, and such a great connoisseur in the art of drawing. The figures of fœtusses which we possess of Froien Albinus, Wrisberg, William Hunter, and Denman, though they are otherwise good, afford no complete representation of an embryo in all the different changes which it undergoes from its first appearance till the fourth and fifth month. What is passing with it after that period, is most masterly represented in the valuable work of William Hunter, which will be considered as an eternal monument of the high degree of perfection the art of engraving graving has reached in England. The present work of Mr. Soemmering is to be next ranked, equalling it with respect to the form in which it is publiſhed, and particularly in the elegance and accuracy of the drawing and engraving. On the firſt plate there are 17 fœtuſſes, figured from the third week after the conception to the firſt half of the fifth month. The ſecond table repreſents in two figures an entire ovum of 5 months, and in the third figure the embryo taken out of it. All theſe figures are drawn with great accuracy in the natural ſhape, and in the very ſituation in which they were obtained. Every change produced by their being kept in ſpirit of wine, the wrinkles, &c. are juſtly omitted. On the title page two ova, one opened, and the other unopened, are likewiſe moſt excellently repreſented. We ſhould wiſh to give an extract of the explanation of the figures, which is interwoven with many new and inſtructive remarks, but we are afraid of exceeding the limits of this Journal, and we only beg leave to ſubjoin the general remarks premiſed to the explanation, as, on account of their novelty and general intereſt, they deſerve to be communicated to our readers. The embryo increaſes very faſt during the firſt days and weeks, but ſlower afterwards, and never in equal proportion; for, in the ſecond month it ſeems to grow ſlower than in the third; in the beginning of the fourth month in a ſtill leſs degree; while, in the middle of that month, it increaſes more till the ſixth, when, about this ti[illegible]e, it begins to grow in a leſs degree till the ninth month. The ovum is proportionably greater and bigger the younger the fœtus is. In the two firſt months the fœtus keeps itſelf in a bent ſituation, ſtretching itſelf afterwards; but towards the end of pregnancy it takes again the former poſture. The head hangs down in every period, bearing always a larger proportion the younger the fœtus is. In very early ones the head exceeds the reſt of the body in largeneſs, and that part which contains the brain has a larger proportion than the face, the younger the fœtus is. The moſt formed parts of the head in the beginning are the oſſa temporum, but of the neck no rudiments can be traced in the third month. The limbs appear like ſmall roundiſh knobs, increaſing and forming in the ſecond month. The eyes, in very ſmall fœtuſſes, ſhew themſelves as two ſmall black dots, the eye-lids open in the ſecond month, or at leaſt are ſo tranſparent that theſe dots are viſible; they ſhut afterwards: the external part of the iris is formed earlier than the inner. The external ear forms in the following progreſſion: At firſt the middle part of the helix is remarked, then the tragus, then the antihelix and the lobe of the ear, and at laſt the ſuperior and inferior part of the helix; and in the fifth month the cornea and ſcapha are formed; however, the total form of the ear is by no means ſo perfect as in an adult. The noſe of a fœtus is likewiſe differently formed. The genitals are from the beginning evidently conſpicuous. The penis is more elongated in very young fœtuſſes, and the glans uncovered. In female fœtuſſes a rima is perceived in lieu of the genitals; and in the third month the clitoris begins to be viſible, and appears prominent till the the fixth month. The umbilical chord is fhort and thick at firft, but grows more in length afterwards. Mr. Soemmering has remarked in feveral well preferved, even very young, fœtuffes the following fexual differences: The thorax is moft ftrikingly different in both fexes. In the male fœtus it is longer, conical, and wider, than the belly and pelvis. The female thorax is fhorter, widens till the fourth rib, where it becomes narrower, and its diftance from the pelvis is greater; female fœtuffes are flat breafted and big bellied. Another mark of difference lies in the head, which is bigger in the male fœtus, but lefs round, &c. The fuperior extremities are longer in a male fœtus; the fhoulder-blades ftronger and prominent, the arms more conical, the fore arm more flefhy, the carpus broader, and the tops of the fingers more pointed. The inferior extremities lie clofer together in a male fœtus, are more flender, and the knuckles and heels are thicker, and the great toe larger than in a female fœtus. There is likewife a fexual difference in the back bone, which however is not expreffible by a figure. Mr. Soemmering concludes by entering into the queftion, whether the fame faculties of the mind and body are innate in men, or whether the different conftruction of the body produces a difference in thofe mental faculties? and he is inclined to affent to the fecond part of the queftion.

---

*Pathologifch Practifhe Abhandlung, i. e.* Pathological and Practical Differtation on the vapours, or wind in the inteftinal tube, intended for Phyficians and invalids. By J. Ch. G. Ackermann, Profeffor at the Univerfity of Altdorf, 1800. Altdorf and Nuernburg, in 8vo. pp. 386, price one rixd. or about 3s. 6d.

Amongst the medical publications that appeared laft Eafter, the prefent deferves a particular notice. Dr. Ackermann treats in the firft chapter of the different gafiform fluids contained in the inteftines; in the fecond, of the preternatural ftate of them; in the third, of the fymptoms that originate from an accumulation of air in the inteftinal canal; in the fourth, on the caufes of the wind or flatus; in the fifth, of the cure; and in the fixth, of what manner of life, perfons ought to obferve for preventing them. Although we have met in the perufal of this book, fome inaccuracies, and although we could wifh that the author had been more copious in its dietetical part, as it is not only devoted to profeffional men, yet we can juftly recommend it as containing a great deal of information, and it may prove equally inftructive to the phyfician as well as to the common reader.

---

### *Profeffor Reich's Theory and Practice of Fever.*

The public have been already informed, through the medium of this Journal, of the difcovery of a new remedy, founded upon a peculiar theory, with which Profeffor *Reich* pretends to cure all fevers, of whatever defcription they may be. This matter engaged the attention of the public ftill more, when the King of Pruffia

Pruffia ordered a committee, confifting of the moft refpectable phyficians of Berlin, to whom the Profeffor was to communicate his arcanum, for inquiring into the nature of that difcovery; and in cafe it fhould in fome meafure be found fatisfactory, the inventor was under the condition of publifhing it for the benefit of the community, and to receive a due reward from the king.

The refult of that inquifition proving, on the whole, not unfavourable to Dr. *Reich*, a pamphlet is now publifhed, in which both the theory and practice of his new method are propofed. It is entitled, "*Gottfried, Chriftian Reich, M. D. und Profeffor, &c. vom Fieber und deffen Behandlung Uberhaupt*; i. e. on Fever, and its Treatment in general; publifhed at the order of His Majefty the King of Pruffia, by the Collegium Medicum et Sanitatis: Berlin, for Frederic Maurer, 1800; pp. 102, 8vo.

We fhall endeavour to give our readers a faithful extract of this publication, upon which the general attention is now fixed, and we leave it to their judgement, whether the principles laid down in it, deferve fo much regard as has been fhewed to them by this public tranfaction under the authority of an enlightened king. The whole is aphoriftically difpofed in paragraphs; and firft, the theory is explained upon which the practice is founded; and then concluded, with marking the advantages which may refult to the art of healing as well as to the community, from this new method of cure.

"All functions of the human body, fecretions as well as excretions, are produced by the continual action of animal chemical proceffes, whereby the mixture of organic matter is perpetually changing. Thefe proceffes originate in the uninterupted reaction of powers or principles, that act in oppofition to one another; and from the reciprocal action of thofe oppofite principles, refults *life*, which confifts *in a continual tendency of heterogeneous matters towards homogeneity;* or, in other words, *in a continual circular motion, produced by the conflict of adverfe principles.* Organization being now a chemical product, its actions or qualities, vis vitalis, incitability, fenfibility, irritability, &c. have a chemical origin; and confequently all actions of fingle parts of the human body, are founded in animal chemical proceffes. The actions of the fluid parts of the human body, owe evidently their origin to thefe proceffes; and as all folid parts may be reduced to fluids, their actions likewife depend on them. It is, however, to be remarked here, that the word *fluid*, as well as that of *chemical*, muft be taken in the moft extenfive fenfe, according to which it comprehends all gafiform fubftances, and even thofe that cannot be reprefented by themfelves, and which we know under the name of magnetic, Galvanic, electric matter, &c. From thefe principles it follows, that all changes of the human organic body, depend on the changes of its chemical qualities, and that even the faculties of the mind are influenced by them, which, however, are able to re-act upon the chemical qualities. The body being expofed now according to the common courfe of nature, to a continual influence of chemical powers, that manifeft a continual tendency to homogeneity, it is only capable of retaining its exiftence by oppofing to this, a tendency to heterogeneity; and as long s thofe chemical potencies do not acquire a furplus, or as long as the animal chemical proceffes proceed under the above condition, the body will retain its identity, i. e. it will continue to live; but as foon as its proceffes are no more produced after animal chemical, but after the dead phyfico-chemical laws, it will ceafe to live, and enter into the power of the common courfe of Nature. The animal chemical proceffes differ from the dead chemical or phyfical, by having their place of action in the animated body, where the common laws of chemical affinity are differently modified.

"The moft important animal chemical proceffes, are *refpiration* and *nutrition*; at the ceffation of either of them, death is the inevitable confequence. Refpiration is the moft neceffary and primitive function of the human conftitution, to which all other functions are fubordinate. The body obtains by refpiration from the atmofpheric *oxygen*, a neceffary principle for its prefervation. Befides this, the atmofphere contains another effential conftituent, *the azote*, and a fmall proportion of *carbon*, all which fubftances are combined and reduced to the confiftency of gafes by the *calorique*. It is known that atmofpherical air is only fit for refpiration by containing *oxygen*, but in fo loofe a ftate, that it eafily feparates in the lungs from its combinations with the other conftituents. Refpiration now is to be confidered as one of the moft fimple animal chemical proceffes, becaufe the combination of the oxygen with the blood during the act of breathing, is effected according to the pure laws of chemical affinity. But when oxygen is only confumed in refpiration, it may be afked, why has Nature provided the atmofphere with fuch a great proportion of azote, that is unfit for refpiration? and why does the atmofphere not merely confift of gas oxygen? This queftion however is eafily anfwered, when we confider that life is founded upon a conteft of adverfe principles, which are here the *azote* and the *oxygen*, both of which muft be looked upon as the proper principles of life. It is highly probable, without any farther proof, from the furplus of the *azote, that this is the ftimulating, or exciting, pofitive principle of life; whereas the oxygen is the tempering, mitigant, limiting, negative principle of life.* The function of the nerves feems to confift in ferving as conductors of thofe principles, an opinion which gains great probability by the Galvanic experiments. The exiftence of the body, however, does not entirely depend on thefe two principles, *azote* and *oxygen*, but it obtains by nutrition other principles, which muft neceffarily be united with thofe, in order to fupport organization. The aliments being diffolved by nutrition into their elementary particles, nutrition deferves with equal right the appellation of an animal chemical procefs, and all fecretions as well as excretions belong to the procefs of nutrition. The different fubftances proper for nutrition, and for this purpofe fecreted in the body, or evacuated from it, are always influenced by the abfolute laws laws of chemical affinity, which are, however, differently modified in the human living body, by feveral circumftances and conditions.

"As long as thefe laws of chemical affinity are thus modified, there is always an equilibrium of the different functions of the human body; this remains in a found ftate: but as foon as the equilibrium is difturbed, and the laws of affinity declining from the vital path, approach to the dead chemical affinity, a tranfition into a difeafed ftate takes place. The fecretions being fubordinate to the procefs of nutrition, experience the fame morbific change, when that procefs is affected in a difeafed manner. This is proved by the products of the fecretions and excretions, that appear more or lefs changed in a morbid ftate, which is particularly evident in fevers, where no excretion or fecretion exifts, which muft not be, or is not changed, viz. urine, alvi excretio, breath, tranfpiration, blood, gall, &c. Fevers, therefore, differ only from a healthy ftate of the body, by the excretions and fecretions being thus changed, that the general equilibrium of the different organs and conftituent particles is difturbed, and confequently a general difproportion in them produced. As the fecretions and excretions in the found ftate, as well as in fever, confift in a feparation and combination of the different fubftances operating in the body, regulated according to the laws of organization, they may be compared with the procefs of fermentation, which goes on naturally and regularly in a found ftate, and is difturbed when the body is difeafed, where at the fame time the general equilibrium is deranged. However, in drawing this comparifon, it muft always be regarded that this fermentation proceeds in a living body, where the common laws of fermentation cannot indifcriminately be applied, but fuffer a change by the organization. Fever therefore is a modification of life, which by its form and generic character, differs from any other modification, which we comprehend under the idea of difeafe. All fevers, from the *ephemera* to the *plague*, the higheft degree of febrile affection, are fpecies of the fame genus, fuppofing they have the fame generic character, or fomething peculiar and effential common to them all, without which they cannot be ranked under the name of fever. This generic character is not materially perceptible, but rather abftrufe, and only noticed by collecting and comparing the phenomena of fever and the perceptible external caufes. We only obferve that every thing which difturbs the juft and general proportion of both principles of life, among one another, or with the other fimple or compound fubftances that the body contains, produces a preternatural fermentation, and excites fuch fymptoms which are afcribed as peculiar to fever. Thefe fymptoms confift in a greater or lefs change of all fecretions and excretions, depending on a difturbed proportion of the fubftances acting on the body, and in it, which is effected on abfolute diminution, or chemical adaptation of oxygen, influenced by certain external circumftances.

"The generic character, or the nature of fever, confifts accordingly *in a preternatural ſeparation and combination of the moſt ſimple conſtituent particles of the human body, effected by a preternatural, abſolute or relative, topical or general, diminution of oxygen.* This diminution of the oxygen is as eaſily occaſioned by external as by internal cauſes. Among the external cauſes may be counted, beſides the common miaſmata and corrupted particles of the atmoſphere, the exanthematous contagia, which when received into the body cauſe a fermentation, whereby the due proportion of oxygen towards the other elements of the body is aboliſhed, and a preternatural ſeparation and re-union of them produced. As the ſound ſtate of the body comprehends a continual ſeries of animal chemical proceſſes, or, in other words, a continual fermentation, ending and beginning with every moment, all external things or circumſtances, interrupting or diſturbing the natural progreſs of theſe proceſſes, are capable of exciting a fever, and the temperature of the air, the ſtate of electricity, have a great influence in this reſpect. The internal potencies which produce a febrile ſtate, are either pre-exiſtent or generated in the body; and every thing that diſturbs the action of the ſolid parts, muſcles, nerves, and veſſels, proceeding according to chemical laws, is able to produce a fever, in which likewiſe mental impreſſions have no ſmall ſhare.

"The proximate cauſe, therefore, of all fevers is grounded either in an obſtructed due reception of oxygen, or in the preternatural adaptation of it, and in the exceſſive accumulation and extrication of azote, hydrogen, carbon, ſulphur, and phoſphorus, and all other elementary particles of the body, and in the various combinations of theſe ſubſtances with one another, or with acceſſory ſubſtances by which they are modified, as light, calorique, magnetic, and electric matter. The phenomena of fever differ according to the ſhare which either of thoſe ſubſtances have in producing the febrile ſtate, according to their particular ſeat of action to the degree and manner of combination, and according to the incitability or activity of the organic parts, which is hereby influenced. When the organiſm has not yet loſt its power or incitability, to re-eſtabliſh the due proportion of thoſe principles, and no organ neceſſary for life is injured or deſtroyed, and the animal chemical proceſſes do not yet too much approach to the dead chemical or phyſical laws, every fever muſt and will ſurely and radically be cured, *by reſtoring the want of oxygen* in ſuch a manner, that no viſcus neceſſary for life is endangered by it.

"*Oxygen* is therefore the only ſure remedy againſt fever, which, though it may differ by its origin, always depends on an abſolute or relative want of it. As this element cannot be exhibited by itſelf, thoſe ſubſtances muſt be employed for the cure of fevers, in which it is contained in the moſt pure and ſimple ſtate,—and theſe ſubſtances are *the acids*. Every acid being the production of a baſis acidifiable and oxygen, an acid is more proper for the cure of fever, the leſs of the former and the more of the latter it contains. On this this account, *the mineral acids* furpafs all other, and are particularly adapted for the purpofe.

*Profeffor Reich* proceeds now to anfwer the objection which may be made to him, that oxygen is too ftrictly adherent to the acidifiable bafis of the acids, to be feparated in the body.—"Every combination of an acid with other fubftances, is a procefs of combuftion, which cannot take place without the combination of the oxygen with the combuftible element. That the combination of an acid with other fubftances, is to be confidered as a procefs of combuftion, appears from the action of ftrong mineral acids upon vegetable and animal fubftances, which are thereby deftroyed; and this combuftion is more vehement and perfect when the acid is ftrong, lefs perceptible when it is weak: oxygen, however, is always difengaged by it. The fame procefs now proceeds in the body, by the acids uniting in the fubftances it meets there; the degree of combuftion differs according to the ftrength of the acid and the general temperature of the body. It is very probable, that even *muriatic acid*, which has not yet been decompofed by the common proceedings of experimental chemiftry, on account of its too great affinity to oxygen, fuffers that decompofition in the human organifm; for falt feems to be very neceffary for mankind, &c.

"Although it feems contradictory to affert, that the exceffive accumulation and extrication of calorique is prohibited in the quickeft and fureft way by mineral acids, and whereas we know by a common experiment, that at the combination of fuch an acid with other fubftances, a great deal of calorique is fet free, yet it may be thus explained: Mineral acids are never given in a pure ftate, and without being previoufly diluted; and thofe fubftances with which they are diluted, are always deprived of a great part of their calorique. If now, at the combination of the oxygen with organic fluidities, the calorique is difentangled, it is immediately imbibed by thofe fubftances which are in want of it, and again bound; it is no more in a free ftate, as it was in the dry febrile heat, but it goes off with the fecretions and excretions, according to the natural progrefs of organifm.

"But if we even abftract from all theoretical reafoning, experience bears alfo evidence to thefe ideas, as acids have been empirically found to be the beft remedies againft fever. Fevers, therefore, are only cured when fuch a quantity of oxygen is brought into the body and equally diftributed in it, as is required for re-eftablifhing the equilibrium amongft its different conftituent particles, which was difturbed by the febrile motions. The Profeffor draws now a nearer comparifon between the procefs of fermentation and a febrile ftate, which, as is abovementioned, he confiders as a fermentative act, in which the common laws of fermentation take place, modified only by organifm. As this is influenced by the temperature and the addition of feveral fubftances, the fame happens in a fever where the reftoration of the equilibrium is in a fimilar way either promoted or difturbed, or preternaturally changed.

The

The progrefs of fever requires, like fermentation, certain periods, before it is accomplifhed, and runs alfo through different ftadia, before the fever is removed by crifes.

"Led by thefe principles, and by the inftinct of febrile patients for the ufe of acids and other fubftances that contain an abundance of oxygen, and induced by former experiments, which proved their efficacy in fevers, Dr. Reich difcovered *the mineral acids* to be the fureft remedies againft fever, and he even applied them with hopes of fuccefs in the laft periods of fevers, where death feemed inevitable; and no phyfician had yet conceived the indication of employing them, nor ever adminiftered them. He thought himfelf the more entitled to entertain hopes of fuccefs, as there is no abfolute danger in fevers; for if we confider life as an animal chemical procefs, it can only be endangered when the general mixture of the conftituents of the body approaches to the higheft degree of animal fermentation, by which the equilibrium is either reftored or entirely fuppreffed by a chemical deftruction of incitability. This degree of animal fermentation enfues either according to the natural and peculiar courfe of that procefs, or is brought on by different external and internal caufes; and the danger of life increafes in proportion to the velocity with which the animal fermentation runs through the different ftadia to the higheft degree.

"It remained now to determine, in what quantity the acids may be given. For this purpofe, Prof. Reich made experiments on himfelf, and began to take fulphuric acid, the internal ufe of which had been long ago recommended; and he affures us, that after having purpofely caufed himfelf an indigeftion, he confumed by degrees in one hour, a whole ounce of concentrated fulphuric acid, which was diluted with a great quantity of water. This large dofe had no other effect, but that the belly was a little inflated, a great quantity of wind went off, and after a reftlefs night, feveral ftools followed."

The firft cafe in which it was made ufe of, is related in his *Cafes of Difeafes,* (in German) Nurnberg, 1800, Vol. I. "Every fymptom of approaching death was prefent here, fingultus, fubfultus tendinum, &c. which he confidered as Galvanic convulfions, produced by the furplus of fubftances oppofite to oxygen. About one hundred drops of concentrated fulphuric acid, diluted with a fufficient quantity of fcrup and water, were adminiftered the firft time; but as a vomiting was brought on by this dofe, the fecond confifted only of fifty drops, and likewife the third, and the remaining two, full dofes of two hundred drops. The abdomen of the patient being much inflated with a great degree of tenfion, a fymptom originating from the generation of different gafes, Dr. Reich determined to try the external application of acids, particularly as he was confcious of the great avail of clyfters of vinegar in any ftate of malignity. For this purpofe, muriatic acid feemed beft to be calculated, becaufe it is milder and more volatile than the fulphuric acid, and eafily combines itfelf in the form of of a gas with other gafes. A clyfter of warm water was accordingly applied, with forty drops of muriatic acid, which, as it produced an evacuation by ftool, with much wind, to the confiderable relief of the patient, it was repeated a fecond time. The fuccefs of thefe two remedies fully anfwered the expectations which had been entertained of them; and the patient got, by the external ufe of fulphuric acid, and the internal ufe of muriatic acid, quite out of danger, after the fpace of two hours, and entirely recovered."

[ To be continued in our next. ]

---

*A Profpect of exterminating the Small-Pox*; *being the Hiftory of the Variolæ Vaccinæ, or Kine-Pox, commonly called the Cow-Pox; as it has appeared in England*; *with an Account of a Series of Inoculations performed for the Kine-Pox, in Maffachufetts.* By BENJAMIN WATERHOUSE, M. D. Fellow of the American Philos. Society; and Profeffor of the Theory and Practice of Phyfic in the Univerfity of Cambridge, 8vo. pp. 40. Bofton, 1800.

We have perufed this very judicious and accurate work with uncommon gratification. The fame temperate and philofophic fpirit which characterized Dr. Jenner's publications, have been caught by Dr. W. and we have no doubt that his countrymen will confider him as the Jenner of the weftern world.

"In the beginning of the year 1799, I received from my friend, Dr. LETTSOM, of London, a copy of Dr. EDWARD JENNER'S "*Inquiry into the caufes and effects of the* VARIOLÆ VACCINÆ, or Cow-Pox;" a difeafe totally unknown in this quarter of the world. On perufing this work, I was ftruck with the unfpeakable advantages that might accrue to this country, and indeed to the human race at large, from the difcovery of a mild diftemper that would ever after fecure the conftitution from that terrible fcourge, the fmall-pox. My attention was not the lefs awakened by a previous impreffion that the fmall-pox came originally from the brute creation; for all that I could recollect of the hiftory of the famous Mahomet, and his *fucceffor*, and of modern Arabia, confpired to ftrengthen the idea that the fmall-pox came to the human race through the brute creation.

"Perceiving that this difeafe began to excite a fpirit of enquiry among our literary men, I deemed it of importance to collect and examine every thing that had or might be publifhed on the fubject, and to acquire, from my correfpondents in England, every information refpecting a diftemper fo interefting to humanity.

"As the great queftion which the profeffional public were anxious to have refolved was, *whether a perfon who had been fairly infected with the genuine* COW *or* KINE-POX, *were thereby fecured against the small-pox*, I bent all my enquiries to afcertain this point.

"It would be fuperfluous to mention every queftion I put, and tedious to relate the different anfwers received. Suffice it for the prefent to fay, that I made my enquiries of the phyficians living in different different parts of Great Britain, and of thofe too who were the leaft fanguine, although moft interefted in the event; of men, who objected much, and believed flowly, yet have in the end become its moft potent advocates. And I do now deliberately declare, that I have received a croud of evidence in confirmation of the doctrine, "that the cow, or kine-pox renders the human frame unfufceptible of the fmall-pox," too great to be refifted by any mind not perverted by prejudice. In truth, the fubject has been traced in England, by thofe who doubted, until conviction became too ftrong for argument, and theoretical objections gave way to ftubborn facts. The confequence has been, that THIRTY THOUSAND perfons, from two weeks old and upwards, have paffed fafely through the difeafe. Dr. JENNER has been particularly noticed by the KING, who gave him permiffion to dedicate the new edition of his book to him.

"But diftance of fpace operates on fome minds like diftance of time. People are not fo ready to believe what happened a great while ago, or a great way off. I therefore found it neceffary to bring the matter home to us, and to repeat in America the experiments performed on the other fide the Atlantic. I wifhed alfo to examine another *important fact*, of which fome eminent phyficians in London expreffed fome doubts, and which I myfelf was anxious to fee more firmly eftablifhed, namely, *whether this new difeafe, this* COW-POX, or KINE-POX, (denominate it which you will) *be really not* CONTAGIOUS, *or catching from one perfon to another.* And I do now affert, that from all the experiments hitherto made public, it clearly appears, that *this fubftitute for the fmall-pox cannot be communicated by any other means than by the actual* CONTACT OF MATTER; or, in other words, *is not catching from one perfon to another by effluvia, like the fmall-pox or meafles.* Even the cows do not convey the diftemper by effluvia, or when there is a fence or hedge interpofed between them; and not, fays Dr. JENNER, unlefs they be handled or milked by thofe who bring the infectious matter with them.

"Having thus traced the moft important facts refpecting the caufes and effects of the *kine-pox* up to their fource in England, and having confirmed moft of them by actual experiment in America, one experiment only remained behind to complete the bufinefs. To effect this, I wrote the following letter to Dr. ASPINWALL, phyfician to the *Small-pox Hofpital* in the neighbourhood of Bofton.

DEAR DOCTOR, *Cambridge, Aug. 2d,* 1800.

"You have doubtlefs heard of the newly defcribed diforder, known in England by the name of the *cow-pox*, which fo nearly refembles the fmall-pox, that it is now agreed in Great-Britain, that the former will pafs for the latter.

"I have collected every thing that has been printed, and all the information I could procure from my correfpondents, refpecting this diftemper, and have been fo thoroughly convinced of its importance to humanity, that I have procured fome of the vaccine matter, matter, and therewith inoculated ſeven of my family. The inoculation has proceeded in ſix of them exactly as deſcribed by WOODVILLE and JENNER; but my deſire is to confirm the doctrine by having ſome of them inoculated by you.

"I can obtain variolous matter, and inoculate them privately, but I wiſh to do it in the moſt open and public way poſſible. As I have imported a new diſtemper, I conceive that the public have a right to know exactly every ſtep I take in it. I write this, therefore, to enquire whether you will, on philanthropic principles, try the experiment of inoculating ſome of my children who have already undergone the cow-pox. If you accede to my propoſal, I ſhall conſider it as an experiment in which we have co-operated for the good of our fellow-citizens, and relate it as ſuch in the pamphlet I mean to publiſh on the ſubject.

I am, &c. &c.

B. W.

Hon. WILLIAM ASPINWALL, Eſq. Brookline.

"To this letter the Dr. returned a polite anſwer, aſſuring me of his readineſs to give any aſſiſtance in his power, to aſcertain *whether the cow-pox would prevent the ſmall-pox;* obſerving, that he had at that time freſh matter that he could depend on, and deſiring me to ſend the children to the hoſpital for that purpoſe. Of the three, which I offered, the Doctor choſe to try the experiment on the boy of 12 years of age, mentioned in page 20, whom he inoculated in my preſence by two punctures, and with matter taken that moment from a patient who had it pretty full upon him. He at the ſame time, inſerted an infected thread, and then put him into the hoſpital, where was one patient with it the natural way. On the 4th day, the Dr. pronounced the *arm* to be infected. It became every hour ſorer, but in a day or two it dried off, and grew well, without producing the ſlighteſt trace of a diſeaſe; ſo that the boy was diſmiſſed from the hoſpital, and returned home the 12th day after the experiment. One fact, in ſuch caſes, is worth a thouſand arguments.

"It is proper to mention, that there are ſome circumſtances, which if not attended to critically, may bring the inoculation of this recently imported diſtemper into a temporary diſrepute. Dr. Jenner, aware of ſuch an accident, has pointed out the fallacious ſources whence a diſeaſe imitative of the *variolæ vaccinæ*, or *kine-pox*, may ariſe, with a view of preventing a ſpurious diſeaſe.

"It was juſt about the ſame time, that Dr. SIMS wrote the letter referred to in page 33; ſo that the flattering proſpect of baniſhing the ſmall-pox for ever from Great Britain, ſeemed to be obſcured for ſeveral weeks. But Dr. JENNER, and Mr. R———; a very diſtinguiſhed ſurgeon and native of the county where the cow-pox firſt appeared, undertook to examine how it happened, that a diſtemper ſo mild in Glocceſterſhire, ſhould be converted into a pretty ſevere diſeaſe in London. This matter was unravelled, and the end of it appeared to be this: The firſt ſubjects inoculated for the kine-pox, were chiefly people maintained as *poor*. They were

 inoculated inoculated at the fmall-pox hofpitals, and feveral of them for both kinds, fmall-pox and kine-pox at the fame time, or at an interval of a day or two, by way of experiment; and it is more than probable, fays one of my correfpondents, that a lancet infected with variolous matter, was ufed for inoculating for the kine-pox. Be that as it may, it is certain that the patients of a celebrated inoculator, had the difeafe with greater feverity than any other practitioner. In general, the patients had more fever, forer arms, and more puftules in London, than in the country. Thofe of the author's friends who have urged him to eftablifh an hofpital for the kine-pox, will now fee more clearly, the reafons for not following their advice.—An hofpital might poffibly heighten a very mild diftemper into a formidable difeafe.

"After this fuccefsful inveftigation, inoculation for the kine-pox went on with redoubled activity; infomuch, that from the date of Dr. Sims' letter, to May following, (juft about a year) 29,400 perfons of all ages, paffed through the difeafe without a fingle death!"

Dr. W. concludes with the following *Poftfcript.*

"Although I am convinced that the kine-pox is a fhorter, fafer, and pleafanter difeafe than the inoculated fmall-pox, even when conducted in the moft fortunate manner, yet there is fome danger of people conceiving too lightly of it. The inoculation of between fifty and fixty perfons of different ages and habits, has taught me, that the kine-pox requires fome care on the part of the patient, as well as attention on that of the phyfician; and I give it as my decided opinion, that an abftinence from animal food and from ftimulating drinks, is as neceffary in the inoculation of the kine as in that of the fmall-pox. A few examples will illuftrate what I wifh to convey. Two young gentlemen were rendered fomewhat uncomfortable for three or four days in confequence of eating and drinking as ufual. One, the leaft attentive to directions, after walking to and from Bofton, in a very hot day, had his febrile fymptoms very much aggravated. His head ach was excruciating, and a flight delirium came on in the evening, with a ftricture acrofs the region of the ftomach, equal to what we fometimes find in the cafual fmall-pox. Such imprudent conduct might have deftroyed him if inoculated for the fmall-pox. A boy, after eating green-corn, was crammed with fruit, under the abfurd idea that ripe fruit can hurt no one, fo that with the fymptoms of kine-pox was joined a cholera morbus. In another boy, the mumps appeared about the 4th day, and arrefted the infection. I could add fome other inftances to prove that this new difeafe, mild and fafe as it is, requires more of the phyfician than merely putting the matter into the arm; but as this treatife is addreffed not fo much to the phyfician, as to the common fenfe of all, I purpofely avoid profeffional directions or criticifms.

*** *We embrace this opportunity of returning our thanks to Dr. Waterhoufe, for his very polite attention to us; and of affuring him, that all hints and propofitions coming from fuch refpectable authority will receive particular attention from us.*—Edit.

*A Conscious View of Circumstances and Proceedings respecting Vaccine Inoculation, &c. &c.* 8vo. pp. 76. *Bath,* 1800.

It is impossible for a candid mind to read this illiberal, and we may say scurrilous pamphlet, without feeling the most lively indignation. We should not have noticed it but for the sake of announcing an answer to it, which is in the press.

